# Successful wire placement into eccentric calcified plaque using intravascular lithotripsy

**DOI:** 10.1186/s43044-025-00656-w

**Published:** 2025-06-05

**Authors:** Hiroshi Abe, Dai Ozaki, Takashi Tokano, Tohru Minamino

**Affiliations:** 1 Department of Cardiovascular Biology and Medicine, Juntendo University Graduate School of Medicine, Tokyo, Japan; 2 Department of Cardiology, Juntendo University Urayasu Hospital, Urayasu, Japan

**Keywords:** Eccentric calcified plaque, Intravascular lithotripsy, PCI

## Abstract

**Background:**

Eccentric calcified lesions pose significant challenges in percutaneous coronary intervention (PCI), as they are associated with an increased risk of coronary artery perforation and suboptimal stent expansion. Moreover, long-term outcomes with drug-eluting stents (DESs) in these lesions are less favorable. Intravascular lithotripsy (IVL) has emerged as a treatment option for calcified lesions. However, its efficacy in managing eccentric calcified lesions remains uncertain.

**Case presentation:**

A 70-year-old male presented with angina starting a week ago. He was diagnosed with unstable angina, and a coronary computed tomography showed severe stenosis with calcified plaque in the right coronary artery. The coronary angiography confirmed severe, calcified, eccentric stenosis in the right coronary artery. Intravascular ultrasound (IVUS) showed an eccentric lesion with calcified plaque, and the diameter of the vessels before and after the lesion was about 6.2 mm on average. Due to the high risk of vessel perforation associated with rotablator and orbital atherectomy systems, intravascular lithotripsy was performed using a 3 mm balloon. The crack formation was observed on IVUS. IVUS image shows both the guidewire and IVUS catheter being partially embedded within the concavity of the calcified nodule, and a 4 mm balloon was used for low-pressure expansion to expand the calcified crackles. This allowed the wire to sink into the calcified plaque and enabled balloon expansion within the calcified region. The risk of coronary perforation was deemed reduced, and a 5 mm × 15 mm DES was successfully placed without complication.

**Conclusions:**

The additional balloon dilation following IVL could allow the wire to enter the eccentric calcified plaque, enhancing procedural safety and effectiveness. Depending on how cracks form within the plaque, this approach may facilitate safer and more effective treatment of eccentric calcified lesions.

## Background

Eccentric calcified lesions pose significant challenges in percutaneous coronary intervention (PCI), as they are associated with an increased risk of coronary artery perforation [1] and suboptimal stent expansion [2]. Moreover, long-term outcomes with drug-eluting stents (DESs) in these lesions are less favorable [3]. Intravascular lithotripsy (IVL) has emerged as a treatment option for calcified lesions. However, its efficacy in managing eccentric calcified lesions remains uncertain.

## Case presentation

**Fig. 1:**
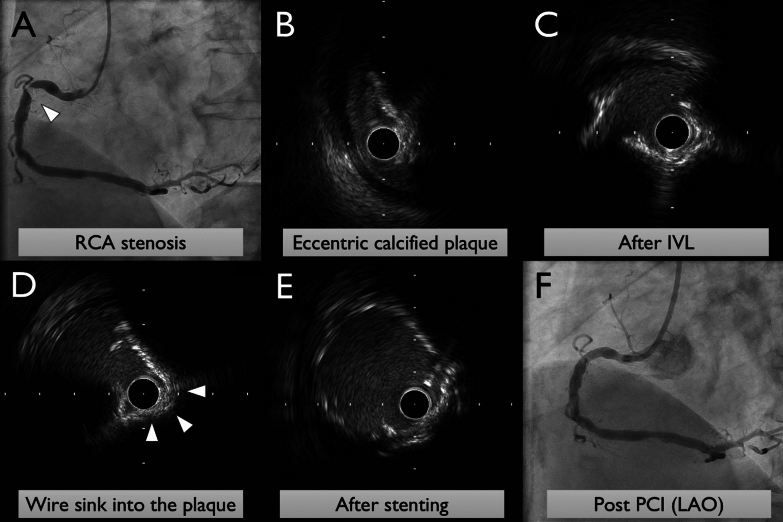
Images of coronary angiography and intravascular ultrasound during primary percutaneous coronary intervention. **A** Coronary angiography (CAG) showing severe, calcified, eccentric stenosis in the right coronary artery. **B** Intravascular ultrasound (IVUS) image reveals the eccentric calcified lesion. **C** The guidewire and IVUS catheter are partially embedded within the concavity of the calcified nodule. **D** IVUS image after expansion using a 4 mm balloon, showing expanded calcified crackles and the wire sinking into the calcified plaque. **E** Successful expansion and placement of a 5 mm × 15 mm drug-eluting stent. **F** Final CAG demonstrated good stent expansion

A 70-year-old male presented with angina starting a week ago. He was diagnosed with unstable angina, and a coronary computed tomography showed severe stenosis with calcified plaque in the right coronary artery. The coronary angiography confirmed severe, calcified, eccentric stenosis in the right coronary artery (Fig. 1A). Intravascular ultrasound (IVUS) showed an eccentric lesion with calcified plaque, and the diameter of the vessels before and after the lesion was about 6.2 mm on average (Fig. 1B). Due to the high risk of vessel perforation associated with rotablator and orbital atherectomy systems, intravascular lithotripsy was performed using a 3 mm balloon, without pre-dilatation using any other balloon. Oversizing may lead to balloon rupture in eruptive calcified plaque, and an undersized balloon aims to ensure emitter contact with the calcified segment. The crack formation was observed on IVUS. IVUS image shows both the guidewire and IVUS catheter being partially embedded within the concavity of the calcified nodule, and a 4 mm balloon was used for low-pressure expansion to expand the calcified crackles (Fig. 1C). This allowed the wire to sink into the calcified plaque and enabled balloon expansion within the calcified region (Fig. 1D). The risk of coronary perforation was deemed reduced, and a 5 mm × 15 mm drug-eluting stent was successfully placed (Fig. 1E) without complications (Fig. 1F).

## Conclusions

The approach for eccentric calcified lesions remains challenging. However, as demonstrated in this case, achieving relatively safe treatment of eccentric calcified plaques may be possible depending on how cracks form within the plaque. The additional balloon dilation following IVL could allow the wire to enter the calcified plaque, enhancing procedural safety and effectiveness.

## Data Availability

No datasets were generated or analyzed during the current study.

## Abbreviations

PCI: Percutaneous coronary intervention
DES: Drug-eluting stent
IVL: Intravascular lithotripsy
IVUS: Intravascular ultrasound
